# Dysregulation of Gap Junction Function and Cytokine Production in Response to Non-Genotoxic Polycyclic Aromatic Hydrocarbons in an In Vitro Lung Cell Model

**DOI:** 10.3390/cancers11040572

**Published:** 2019-04-23

**Authors:** Deedee Romo, Kalpana Velmurugan, Brad L. Upham, Lori D. Dwyer-Nield, Alison K. Bauer

**Affiliations:** 1 Department of Environmental and Occupational Health, University of Colorado Anschutz Medical Campus, Aurora, CO 80045, USA; deedee.romo@ucdenver.edu (D.R.); Kalpana.velmurugan@ucdenver.edu (K.V.); 2 Department of Pediatrics and Human Development, Michigan State University, East Lansing, MI 48824, USA; Brad.Upham@hc.msu.edu; 3 Department of Pharmaceutical Sciences, University of Colorado Anschutz Medical Campus, Aurora, CO 80045, USA; lori.nield@ucdenver.edu

**Keywords:** epithelial cells, gap junctions, inflammation, lung, macrophages, polycyclic aromatic hydrocarbons, tumor necrosis factor (TNF), tumor promotion

## Abstract

Polycyclic aromatic hydrocarbons (PAHs), prevalent contaminants in our environment, in many occupations, and in first and second-hand smoke, pose significant adverse health effects. Most research focused on the genotoxic high molecular weight PAHs (e.g., benzo[*a*]pyrene), however, the nongenotoxic low molecular weight (LMW) PAHs are emerging as potential co-carcinogens and tumor promoters known to dysregulate gap junctional intercellular communication (GJIC), activate mitogen activated protein kinase pathways, and induce the release of inflammatory mediators. We hypothesize that inflammatory mediators resulting from LMW PAH exposure in mouse lung epithelial cell lines are involved in the dysregulation of GJIC. We used mouse lung epithelial cell lines and an alveolar macrophage cell line in the presence of a binary PAH mixture (1:1 ratio of fluoranthene and 1-methylanthracene; PAH mixture). Parthenolide, a pan-inflammation inhibitor, reversed the PAH-induced inhibition of GJIC, the decreased CX43 expression, and the induction of KC and TNF. To further determine the direct role of a cytokine in regulating GJIC, recombinant TNF (rTNF) was used to inhibit GJIC and this response was further enhanced in the presence of the PAH mixture. Collectively, these findings support a role for inflammation in regulating GJIC and the potential to target these early stage cancer pathways for therapeutics.

## 1. Introduction

Polycyclic aromatic hydrocarbons (PAH) are abundant toxicants in the environment (air, water, soil), occupational settings, and in mainstream smoke, secondhand (sidestream) smoke (SHS), and thirdhand smoke exposures [1,2,3,4]. The International Agency for Research on Cancer (IARC) listed SHS and air pollution as group 1 carcinogens in 2012 and 2013, respectively, and the World Health Organization (WHO) recently declared that “air pollution is the new tobacco” with ~92% of the world’s population living in regions that exceed WHO air quality limits [5,6,7]. In addition, the fact that climate changes are predicted to lead to increased air pollutants (e.g., particulate matter (PM)) from events such as wildfires [8] supports a need to understand the composition of these mixtures, which includes many PAHs, and how these mixtures influence the development of diseases, such as cancer.

Previous research on the adverse health effects of PAHs, particularly cancer, focused on the genotoxic high molecular weight (HMW) congeners (≥5 rings), such as benzo[*a*]pyrene (B[*a*]P). B[*a*]P is a known group 1 carcinogen according to the IARC [9,10], while other genotoxic HMW PAHs are primarily classified as group 2B, “possibly carcinogenic to humans”. However, low molecular weight (LMW; 2–4 rings) PAHs are more abundant than HMW PAHs, such as B[*a*]P, in primary and SHS smoke from cigarettes and marijuana (e.g., fluoranthene), in air pollution as components of particulate matter, as well as potential occupational hazards for those workers in industries such as coal, coke, and steel [1,2,3,9,11,12,13,14,15,16,17]. For example, in SHS, the level of 2–4 ring LMW PAHs is ~20 times that of the HMW PAHs [2] and in the Kentucky 1R1 reference cigarette smoke condensates, the methylated anthracenes (LMW PAHs) are 62 times higher than B[*a*]P and benzo(*e*)pyrene [11]. Additionally, many of these LMW PAH species are considered U.S.E.P.A. priority PAHs based on their potential for human exposure and abundance at hazardous waste sites, environmental disasters (e.g., Deepwater Horizon oil spill), among others [3,18]. LMW PAHs, such as fluoranthene, are also prevalent in diesel exhaust (DE), indoor pollution from cook stoves and mosquito coils, and were far more abundant than HMW species at oil production sites [19,20,21,22,23,24]. Given the lack of studies, LMW PAHs are not currently classified as carcinogens (IARC group 3, “not classifiable as the carcinogenicity in humans”), with the exception of naphthalene. Research on the potential adverse health effects, such as cancer, of LMW PAHs in lung is sparse. Therefore, our objective is to provide mechanistic-based empirical evidence to improve the assessment of health risks from LMW PAHs, and potentially identify new therapeutic targets.

Environmental lung carcinogenesis is in general a multi-stage process that begins with the initiation stage, where the presumed precursor cells of lung adenocarcinoma (alveolar type II cells and bronchiolar club cells) undergo a genotoxic event, followed by the tumor promotion stage where there are key components that lead to cellular transformation [25,26,27]. Several of these key components or phenotypes of promotion are also considered either hallmarks of cancer or enabling characteristics, according to Hanahan and Weinberg [28]. Gap junctional intercellular communication (GJIC) falls under the evasion of growth suppression hallmark, specifically during the early stages of tumor development [29]. Gap junctions, composed of connexins (CX), are intercellular channels that allow for molecular communication among neighboring cells [30] and when the communication is altered, impairment of function can lead to pathological states, such as cancer [31,32,33]. However, little is known about their function in the early development of lung cancer or inflammation, with the exception of a few key studies [34,35,36,37,38,39]. Inflammation during promotion is considered an enabling characteristic and is associated with triggering growth of the epithelial cells via production of inflammatory mediators. Thus, we used GJIC and inflammatory mediator production as phenotypes to link the LMW PAHs to promotion based on results from our previous published works [35,36,37,40]. These published results all used an environmentally relevant binary mixture of LMW PAHs (1-methylanthracene and fluoranthene) that affected the following four biological endpoints in mouse lung epithelial cells. (1) Activation of p38 MAP kinase (MAPK), (2) dysregulation of GJIC, (3) induction of gene expression for inflammatory mediators, such as COX2, and (4) when combined with B[*a*]P demonstrated increases in benzo[*a*]pyrene-7, 8-dihydrodiol-9, 10-oxide (BPDE, a major B[*a*]P metabolite) resulting in DNA adducts [35,36,37,40].

We hypothesize that inflammatory mediators resulting from LMW PAH exposure in mouse lung alveolar type II cell lines (C10 and E10) are involved in the dysregulation of GJIC. To test this hypothesis, we used pharmacologic inhibitors of inflammation, including a pan-inflammation inhibitor (parthenolide; NFκB, AP-1, and inflammasomes), COX1/2 inhibitor (indomethacin), glyburide (NALP3 inflammasome inhibitor), and specific inhibitors of NFκB (ACHP) and TLR4 (Cli-095) to evaluate their impact on GJIC activity, and hence function, and CX43 protein expression, the primary CX in the lung [41,42,43]. In addition, we measured the production of chemokine C-X-C motif ligand 1 (KC), a key chemokine involved in the recruitment of neutrophils, an important cell type during lung tumor promotion [44,45], and other inflammatory mediators important in early preneoplastic stages, such as TNF [46], using RTPCR analysis in the epithelial cells. We also used a mouse alveolar macrophage cell line (MHS) to determine the effects of these LMW PAHs on another critical inflammatory cell type during promotion [47,48,49] and measured cytotoxicity and inflammatory mediator production via RTPCR. Lastly, based on the production of TNF by both cell types (epithelial and macrophage), we determined if recombinant TNF would influence GJIC and CX43 expression in the presence or absence of LMW PAHs. Collectively, these studies provide a novel link between GJIC and inflammation in the lung cells that are exposed to an environmental toxicant that is likely involved in tumor promotion and provide new data for future risk assessment.

## 2. Results

### 2.1. Cytotoxicity of the LMW PAHs in Lung Epithelial Cells and Macrophages

**Figure 1:**
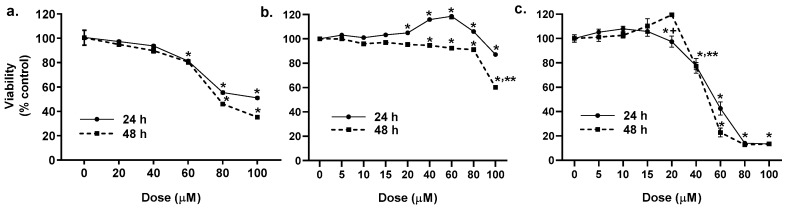
Cytotoxicity for epithelial (C10, E10) and macrophage (MHS) cell lines in response to LMW PAH exposure. In (**a**) C10 cells, (**b**) E10 cells, and (**c**) MHS cells for a dose response with the binary LMW PAH mixture (1:1 ratio of 1-methylanthracene and fluoranthene). Cytotoxicity was performed using an MTS assay at 24 and 48 h of treatment in cells that are serum deprived prior to treatment. For C10 and E10 cells, * *p* < 0.05 for treatment compared to control (DMSO); ** *p* < 0.05 100 μM dose compared to 40–80 μM dose for C10 cells. MHS cells: *, *p* < 0.05 treatments compared to control. + *p* < 0.05 20 μM compared to 40 μM; ** *p* < 0.05 40 μM compared to all other doses.

A dose response for the binary PAH mixture was done for all three cell lines ranging from 0–100 μM (Figure 1), based on previous studies [36,37]. In the C10 cells, the PAH mixture was not cytotoxic up to 60 μM, thus all subsequent studies were done at non-cytotoxic doses below 60 μM (Figure 1a). Cytotoxicity was not observed in the E10 cells at 24 h, however, at 48 h, 40–80 μM was slightly yet significantly toxic and >80 μM was substantially toxic (Figure 1b). In the MHS cells, cytotoxicity was observed at 40 μM, thus doses ≤20 μM were used for the remainder of the studies in the macrophage cell line (Figure 1c). For all remaining epithelial cell studies, doses used were based on gap junction inhibition IC_50_ (C10 cells, 40 μM prior to 24 h and 15 μM at 24 h), previously determined [37], which are noncytotoxic doses.

### 2.2. PAH-Induced Cytokine and Chemokine mRNA Expression in C10 Cells

**Figure 2:**
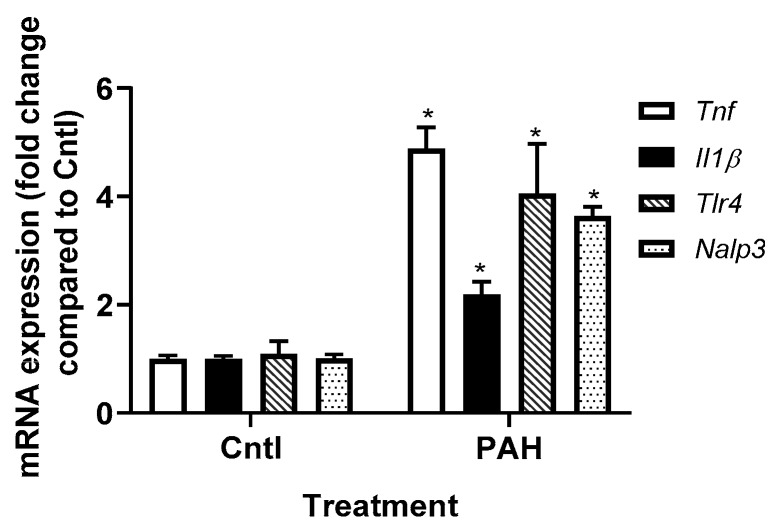
Expression of inflammatory mediator transcripts is increased in response to LMW PAH treatment in C10 cells. C10 cells following 4 h exposure to the PAH mixture (40 μM; 1:1 ratio of 1-methylanthracene and fluoranthene) were then evaluated using qRT-PCR. * *p* < 0.05 PAH treatment compared to control (DMSO). *Tnf*, tumor necrosis factor; Il1β, interleukin 1β; *Nalp3*, NACHT, LRR and PYD domains-containing protein 3 (NLRP3); *Tlr4*, toll-like receptor 4. Experiments repeated 2 times; *n* = 3 per treatment per experiment.

We previously showed in our laboratory that the binary LMW PAH mixture used in these studies induces *Kc*, *Il6*, *Mcp1*, and *Cox2* (Ptgs2) mRNA expression in C10 cells following 4 h of treatment [37]. In Figure 2, we further demonstrate that *Tnf*, *Il1β*, *Nalp3* (NLRP3), and *Tlr4* transcripts are also significantly upregulated in response the same PAH mixture in the C10 cells. Because of these findings and the clear role of gap junctions in the mechanisms driving this LMW PAH-induced toxicity observed previously [36,37,40,50], we evaluated the effects of these inflammation pathways on GJIC using specific pharmacological inhibitors of inflammation in C10 and E10 epithelial cells.

### 2.3. PAH-Induced Inhibition of Gap Junction Activity Is Prevented in Epithelial Cells in Response to a Pan-Inflammation Inhibitor

**Figure 3:**
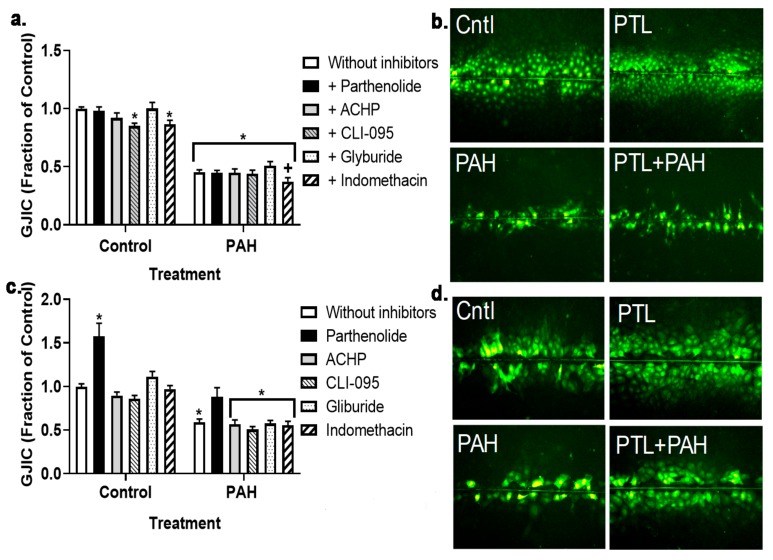
Gap junction intracellular communication (GJIC) dysregulation in response to LMW PAHs is not changed in response to anti-inflammatory inhibitors at an early time point (4 h). (**a**) C10 cells were treated with the binary LMW PAH mixture (40 μM; 1:1 ratio of 1-methylanthracene and fluoranthene) for 4 h following 1 h pre-incubation with these inhibitors (parthenolide, 10 μM); ACHP, 1 μM; CLI-095, 3 μM; glyburide, 50 μM; and indomethacin, 1 μM). (**b**) Representative images of C10 cells following the SL/DT assay used to quantify the gap junction activity in these cells in response to the PAHs and parthenolide (all other inhibitor combinations shown in Figure S1). (**c**) E10 cells were treated the same as the C10 cells with these inhibitors (parthenolide, ACHP, CLI-095, glyburide, and indomethacin). * *p* < 0.05 inhibitors or inhibitors + PAH treatment compared to control (DMSO); +, *p* < 0.05 indomethacin + PAH treatment compared to PAH without inhibitors (white bar on right). (**d**) Representative images of E10 cells following the SL/DT assay in response to the PAHs and parthenolide (all other inhibitor combinations shown in Figure S1). Experiments were repeated 3 times; *n* = 3 per treatment per experiment. PTL, parthenolide. Magnification 100×.

**Figure 4:**
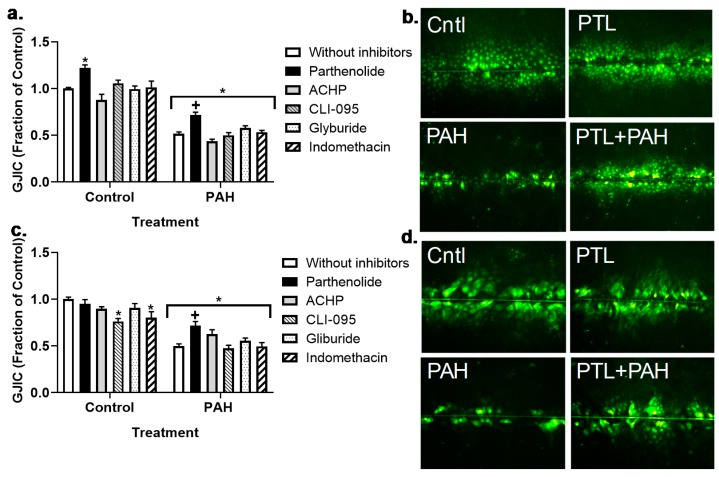
Gap junction intracellular communication (GJIC) dysregulation in response to LMW PAHs is reversed with parthenolide treatment at 24 h in both C10 and E10 cells. (**a**) C10 cells were treated with the binary LMW PAH mixture (15 μM at 24 h; 1:1 ratio of 1-methylanthracene and fluoranthene) following a 1 h pre-incubation with these inhibitors (parthenolide, 10 μM; ACHP, 1 μM; CLI-095, 3 μM; glyburide, 50 μM; indomethacin, 1 μM). (**b**) Representative images of C10 cells following the SL/DT assay in these cells in response to the PAHs and parthenolide. All other inhibitor combinations can be found in Figure S2. (**c**) E10 cells were treated the same as C10 cells in a with these inhibitors parthenolide, ACHP, CLI-095, glyburide, indomethacin. (**d**) Representative images of E10 cells following the SL/DT assay in response to the PAHs and parthenolide. All other inhibitor combinations are in Figure S2. Experiments were repeated 3 times; *n* = 3 per treatment per experiment. PTL, parthenolide. * *p* < 0.05 for treatments compared to the control (DMSO) without inhibitors. + *p* < 0.05 for parthenolide + PAH treatment compared to PAH treatment without inhibitors. Magnification 100×.

**Figure 5:**
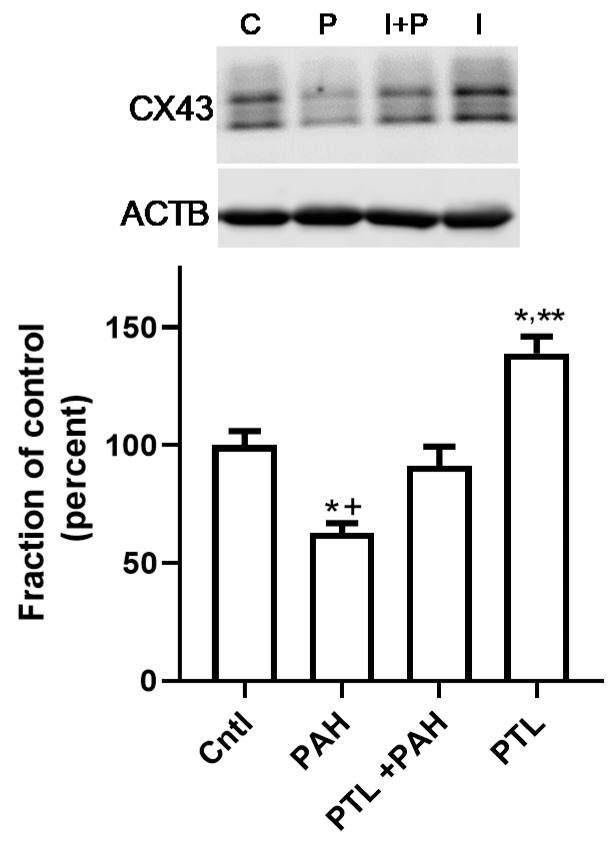
CX43 protein is not suppressed by LMW PAH exposure in the C10 cells in the presence of PTL. C10 cells were pre-treated for 1 h with 10 μM parthenolide (PTL) prior to treatment with the LMW PAH mixture (15 μM; 1:1 ratio of 1-methylanthracene and fluoranthene) for 24 h. Experiment repeated 4 times; *n* = 3 per treatment per experiment. A representative CX43 immunoblot is depicted above the graph (* *p* < 0.05 for treatment compared to control), PAH treatment and PAH treatment in the presence PTL (^+^ *p* < 0.05 for PAH compared to PAH plus PTL), and PTL alone (** *p* < 0.05 PTL compared to control). CX43 protein expression (upper blot) is normalized to β actin (ACTB, lower blot). C, cntl, control (DMSO); P, PAH, LMW PAH mixture; I, PTL, parthenolide (inhibitor); I + P, parthenolide plus PAH treatment.

We previously established that this binary PAH mixture inhibited GJIC in C10 cells at both the 4 h (early) and 24 h (late) time points in C10 cells, and that these PAHs elicited upregulation of cytokines and chemokine mRNA expression. Thus, to investigate potential pathways involved in these mechanisms, we inhibited several inflammation pathways at both time points in cells treated with 40 or 15 μM binary PAH mixture, respectively. Using the MTS cytotoxicity assay, we not observe any toxicity at the inhibitor doses used in any of the cell lines (Bauer, A.K.; Romo, D. University of Colorado, Anschutz Medical Campus, Aurora, CO, USA, 2019). At 4 h, we did not observe any significant effects of the anti-inflammatory compounds on GJIC inhibition in C10 (Figure 3a,b) or E10 cells (Figure 3c,d); see Figure S1. Although in the E10 cells, parthenolide in combination with PAHs was close to significant (*p* < 0.08) and the image in Figure 3d supports this finding compared to the PAH treatment without PTL. However, at the 24 h time point, parthenolide reversed PAH-induced inhibition in C10 and E10 cells while no other compounds had an effect (Figure 4a–d; see Figure S2). In addition, the CX43 protein at 24 h was significantly decreased in the C10 cells in response to the binary PAH mixture (15 μM), however parthenolide also significantly reversed the effect of the PAHs on CX43 protein expression (Figure 5).

### 2.4. Chemokine Upregulation in Response to LMW PAHs: Inhibition with Anti-Inflammatory Compounds

**Figure 6:**
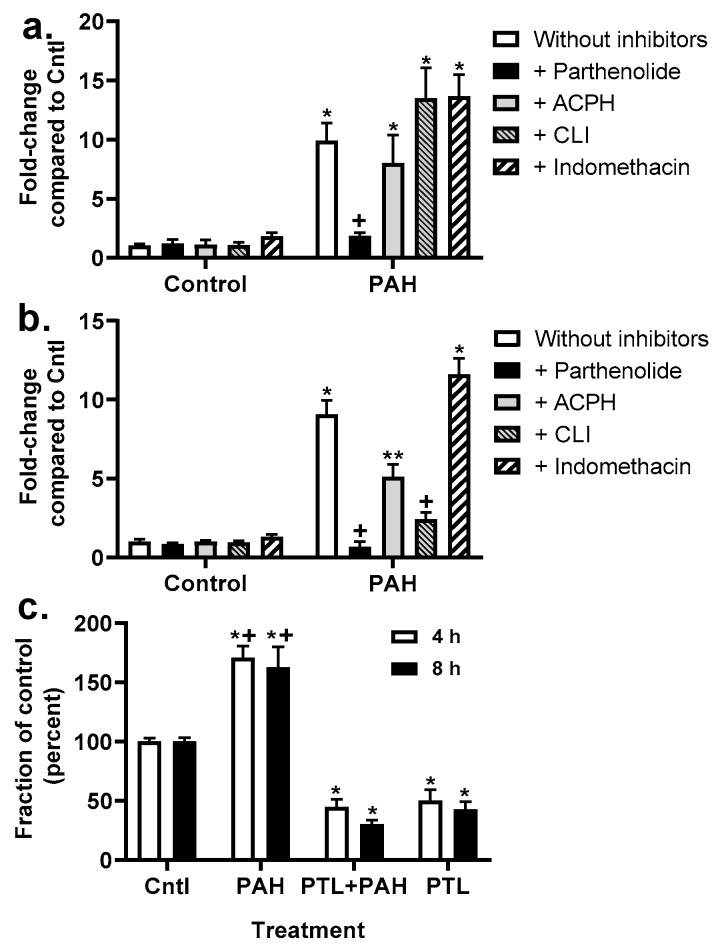
KC transcript and protein levels are elevated in response to LMW PAHs and inhibited in the presence of PTL. *Kc* transcript levels in C10 (**a**) and E10 (**b**) cells in response to 4 h PAH exposure (40 μM 1:1 ratio of 1-methylanthracene and fluoranthene) ± inhibitors. For (**a**,**b**), * *p* < 0.05 for treatment compared to control (DMSO); + *p* < 0.05 for PTL or CLI + PAH compared to PAH without inhibitors; ** *p* < 0.05 for ACHP+PAH compared to PAH without inhibitors. Experiments repeated 2–3 times with an *n* = 3 per treatment. (**c**) KC proteins levels were measured (ELISA) at 4 and 8 h following PAH mixture in C10 cells ± PTL (parthenolide) (10 μM). Experiments repeated 3 times with an *n* = 3 per treatment per experiment.

*Kc* transcript expression was determined after 4 h exposure to the PAHs at the same doses used for the GJIC experiment and revealed that only parthenolide inhibited PAH-induced *Kc* expression in C10 cells (Figure 6a). However, in E10 cells (Figure 6b), parthenolide, ACHP, and CLI-095 all inhibited PAH-induced *Kc* expression. As a result, we then measured secreted KC via ELISA in media from C10 cells exposed to parthenolide in the presence of the binary LMW PAH mixture. The PAHs significantly increased KC protein secretion, while parthenolide inhibited the PAH-induced increase in KC at 4 and 8 h time points (Figure 6). Additionally, *Tnf* transcripts were measured in both cell lines (Figure S3). *Tnf* was significantly induced in response to the LMW PAHs in both cell lines, although to a greater extent in the E10 cells, compared to controls. However, parthenolide significantly inhibited PAH-induced *Tnf* in both C10 and E10 cells.

### 2.5. Production of Cytokines and Chemokines in MHS Cells in Response to LMW PAHs: Effects of Anti-Inflammatory Compounds

**Figure 7:**
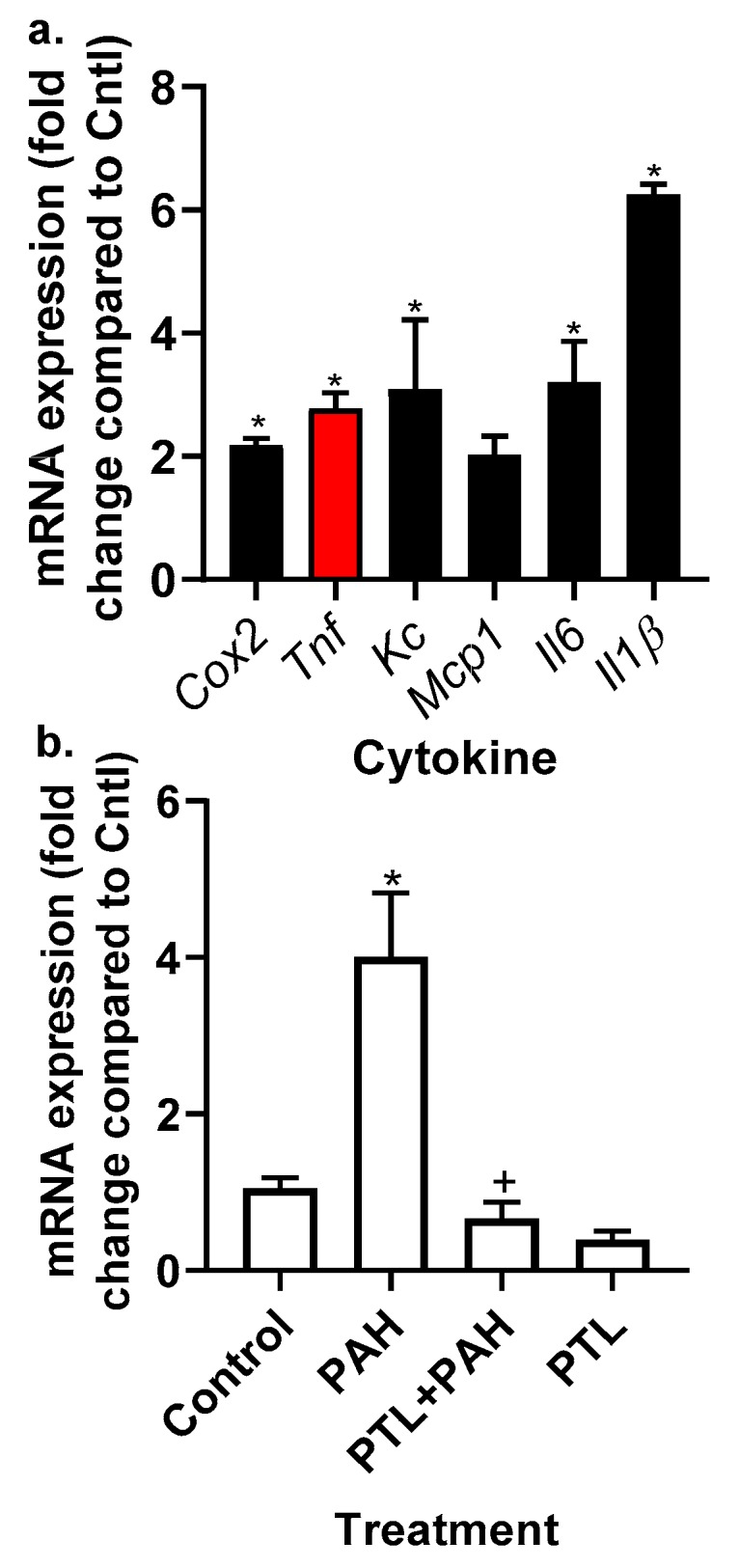
Inflammatory mediator gene expression was elevated in the MHS cells in response to the LMW PAH mixture. (**a**) In response to the 20 μM LMW PAH mixture (1:1 ratio of 1-methylanthracene and fluoranthene), *Cox2*, *Tnf*, *Kc*, *Mcp1*, *Il6*, and *Il1β* were all significantly elevated (* *p* < 0.05) compared to controls (DMSO) cells following 4 h of treatment in the MHS cells. mRNA for each cytokine gene was normalized to 18S and then fold change determined compared to control. Cox2, cyclooxygenase 2; Mcp1, monocyte chemo-attractant protein 1; IL6, interleukin 6. Tnf (red bar) indicates the cytokine we used for the recombinant cytokine studies that follow. (**b**) PTL inhibits *Tnf* expression in response to PAH exposure. * *p* < 0.05 PAH compared to control; + *p* < 0.05 PTL + PAH compared to PAH treatment. Experiments repeated 2–3 times with an *n* = 3 per experiment.

MHS, an alveolar macrophage cell line, was used to examine the production of cytokines and chemokines in response to the binary PAH mixture in an inflammatory cell type frequently observed in response to pulmonary insults. We chose cytokines and chemokines that we previously observed in response to these PAHs in epithelial cells (C10 cells) and that are reflective of acute inflammatory responses [51,52]. The MHS cells were treated for 4 h with several non-cytotoxic doses of the binary PAH mixture (0–20 μM) followed by quantitative RT-PCR (qRT-PCR) analysis; Figure 7a demonstrates the responses at the 20 μM dose and Table 1 the responses at all doses of the binary PAH mixture. *Cox2* and *Tnf* mRNA expression significantly increased in response to the PAH mixture at both 15 and 20 μM doses, whereas *Kc* and *Il6* increased at 20 μM only. However, *Il1β* significantly increased from 10–20 μM doses. *Mcp1* was not significantly changed in response to any of the doses in MHS cells. We then determined if parthenolide could inhibit *Tnf* expression following PAH treatment (Figure 7b). Parthenolide significantly inhibited *Tnf* expression following PAH treatment, further supporting Tnf use in recombinant cytokine studies. In addition, PAH-induced Kc mRNA expression was also significantly reduced in response to parthenolide in MHS cells.

### 2.6. Recombinant TNF (rTNF) Elicits GJIC Inhibition Alone and in Response to Combinations of rTNF and PAHs

**Figure 8:**
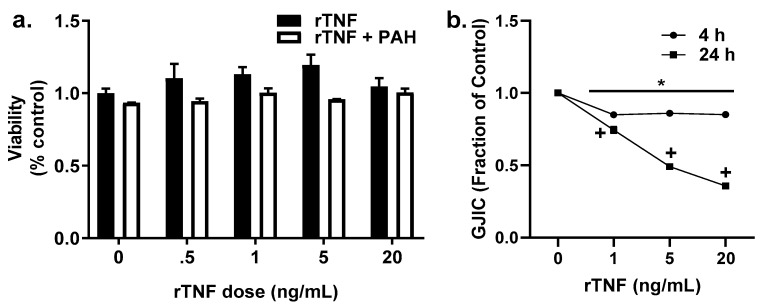
Recombinant TNF (rTNF) is not cytotoxic to C10 cells but does inhibit GJIC. (**a**) Cytotoxicity in C10 cells was assessed using the MTS assay at 24 h to determine any rTNF-induced toxicity or toxicity due to rTNF in the presence to 15 μM LMW PAH mixture (1:1 ratio of 1-methylanthracene and fluoranthene). No cytotoxicity was observed. (**b**) GJIC was measured using the SL/DT assay at both 4 and 24 h time points with rTNF alone. * *p* < 0.05 rTNF treatment compared to control; ^+^ *p* < 0.05 rTNF at 24 h compared to 4 h. rTNF, recombinant TNF; PAH, LMW PAH mixture described above. Experiments repeated 3 times with an *n* = 3 per treatment per experiment.

We then investigated the possibility that a cytokine, TNF, could influence gap junction dysfunction based on our previous studies [36]. These results herein with inhibitors of inflammation provided further evidence that the effects of TNF were downstream of NFκB and additional pathways (see Figure 2, Figure 7 and Figure S3) in this lung cell model for both the epithelial cells and macrophages. In addition, one study using WB rat liver epithelial cells demonstrated that TNF inhibited GJIC [53]. We first demonstrated that TNF in the presence or absence of 15 μM binary PAH mixture was not cytotoxic (Figure 8a). We then evaluated the ability of TNF to inhibit GJIC in C10 epithelial cells. rTNF increasingly inhibited GJIC between the doses of 1 to 20 ng/mL at 24 h (Figure 8b). At 4 h, rTNF significantly inhibited GJIC at all doses, albeit only a 15% reduction as compared to a 25% reduction in gap junction activity using the lowest dose (1 ng/mL rTNF) at 24 h. Thus, we chose to do all following experiments at 24 h.

**Figure 9:**
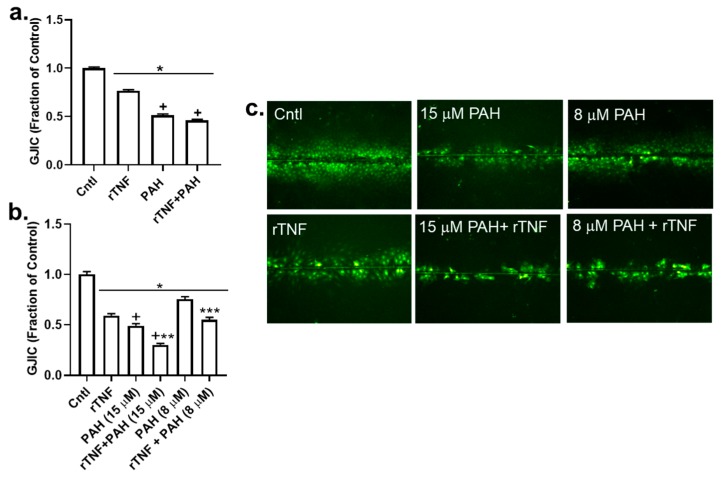
rTNF-induced dysfunction of GJIC was further enhanced in the presence of LMW PAHs. (**a**) A SL/DT assay was used to measure GJIC 4 h following treatment with rTNF (1 ng/mL), LMW PAH mixture (40 µM; 1:1 ratio of 1-methylanthracene and fluoranthene), or combinations of both rTNF and PAH mixture. * *p* < 0.05 treatment at both time points compared to control. * *p* < 0.05 treatment at both time points compared to control; ^+^ *p* < 0.05 for PAH or rTNF plus PAH (40 μM) treatment. (**b**) A SL/DT assay was used to measure GJIC 24 h following treatment with rTNF (1 ng/mL), LMW PAH mixture (8 or 15 μM; 1:1 ratio of 1-methylanthracene and fluoranthene), or combinations of both rTNF at either the low (8 μM) or high (15 μM) dose of LMW PAHs. * *p* < 0.05 treatment at both time points compared to control; ^+^ *p* < 0.05 rTNF treatment at 24 h compared to PAH or the combination of PAH plus rTNF (15 μM) at 24 h; ** *p* < 0.05 rTNF plus PAH (15 μM) treatment at compared to PAH (15 μM) at 4 h; *** *p* < 0.05 PAH plus rTNF (8 μM) at 24 h treatment compared to rTNF or PAH (8 μM) at 24 h. Experiments repeated 3 times with an *n* = 3 per treatment per experiment. (**c**) Representative images of the experiment depicted in (**b**). rTNF, recombinant TNF; PAH indicates the LMW PAH mixture described above. Magnification 100×.

**Figure 10:**
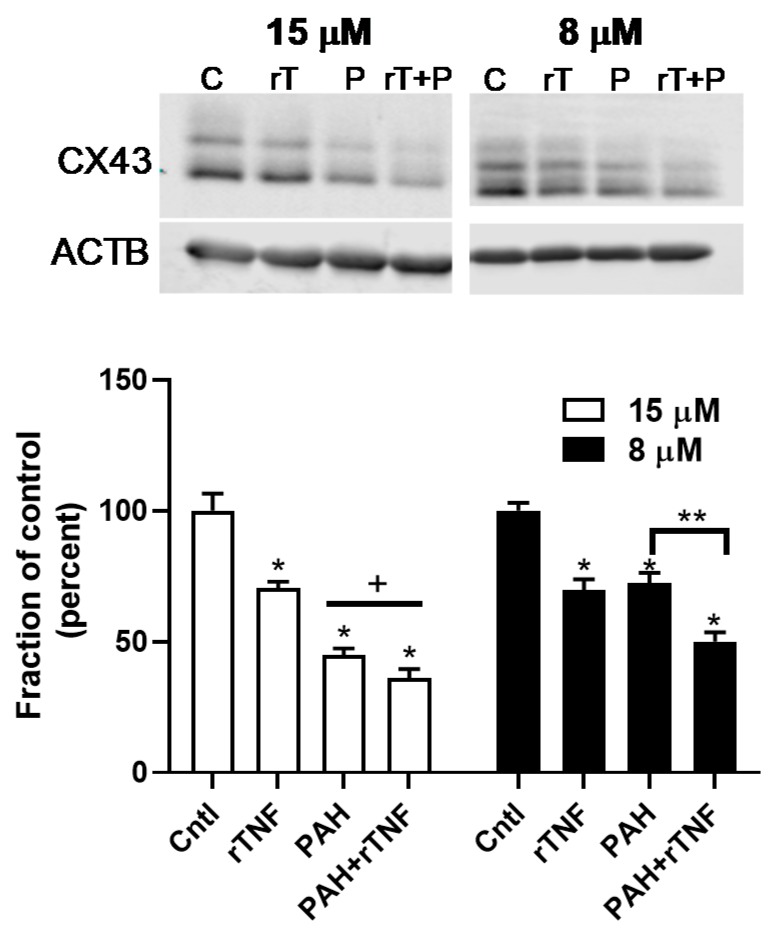
rTNF-induced repression of CX43 protein expression at 24 h is enhanced following co-exposure to rTNF and the lower LMW PAH dose (8 μM). Both the high (15 μM) and low (8 μM) doses of the LMW PAH mixture (1:1 ratio of 1-methylanthracene and fluoranthene) were used to evaluate the effect of rTNF on CX43 protein expression using immunoblots. CX43 protein expression (upper blot) is normalized to bactin (lower blot). rTNF (1 ng/mL), LMW PAHs at both doses, and the combinations of both doses with rTNF are shown. The immunoblot depicted at the top is representative of 3 experiments with an *n* = 3 per treatment group per experiment. * *p* < 0.05 all treatments compared to the control (DMSO); ^+^ *p* < 0.05 rTNF compared to 15 μM PAH or rTNF plus 15 μM PAH; ** *p* < 0.05 8 μM PAH plus rTNF compared to either rTNF or 8 μM PAH alone. C, Cntl, control (DMSO); rTNF, rT, recombinant TNF (1 ng/mL); PAH, P, LMW PAH mixture; rT + P, rTNF plus PAH treatment.

Next, we determined if rTNF would increase the inhibitory effects of the PAH mixture on GJIC. We used a very low dose of rTNF (1 ng/mL) added to the binary PAH mixture (8 or 15 μM), which are doses that mimic the lung microenvironment following exposure to PAHs. As observed in Figure 9A, the rTNF added to the 40 μM PAH mixture did not significantly alter inhibition of GJIC at 4 h as compared to the 40 μM PAH mixture containing no rTNF. However, at 24 h the addition of rTNF to the PAH mixture significantly increased inhibition of GJIC, as compared to the PAH mixture without rTNF (Figure 9B). To obtain a more robust response of this rTNF effect, we used ~1/2 the dose of the PAH mixture (8 μM) and repeated the study at the 24 h time point (Figure 9B, right). The addition of 1 ng/mL rTNF to 8 μM of the PAH mixture, significantly increased the inhibition of GJIC above that observed with the PAH mixture alone, supporting the concept of a toxicant/inflammatory mediator combination effect in the lung. Lastly, we determined if this rTNF effect not only affected gap junction function but also the level of the gap junction protein, CX43 (Figure 10). As expected, at the higher PAH level, the CX43 protein was repressed, but not significantly different from the PAH alone (Figure 10 left), whereas at the lower PAH dose, the level of CX43 repression significantly differed from the PAH and rTNF alone (Figure 10 right). Thus, lower levels of PAH in combination with a cytokine led to significant effects on gap junctions, a biomarker for tumor promotion.

## 3. Discussion

**Figure 11:**
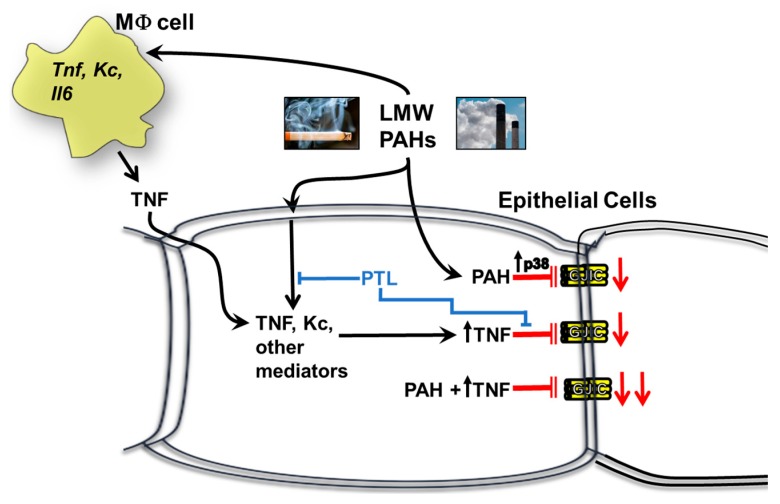
A schematic demonstrating the effects of inflammation on GJIC. PAHs activate MAP kinase pathways, such as p38 MAP kinase and inflammatory mediator pathways, such as cytokines (TNF, KC, and others). Some of these pathways then lead to the inhibition of GJIC. Blue indicates inhibition with PTL. Red indicates inhibition of gap junctions. MΦ, macrophage; PTL, parthenolide; p38, p38 MAP kinase.

Our overall hypothesis for the studies herein was that inflammatory mediators resulting from treatment with these environmentally relevant LMW PAHs negatively influence GJIC in lung epithelial cells. We used both epithelial and macrophage cell lines to show that these PAH-induced cytokines and chemokines not only originate from the epithelial cells, but also the lung macrophages, at PAH doses that are non-cytotoxic. This finding is important given that the lung microenvironment is so complex, and inflammation is involved in the early stages of cancer development that is typically dependent on immune cells, particularly the macrophages [28]. Due to our recent study implicating eicosanoid pathway involvement [49], we determined which key inflammation pathways were involved in PAH-mediated dysregulation of GJIC using several pathway specific pharmacological inhibitors. Namely, cells were preincubated with (1) parthenolide, a pan- inflammation blocker that inhibits NFκB, AP1, and NALP3 inflammasome pathways [54,55]. (2) ACHP that inhibits IκB preventing the activation of NFκB, which is a transcription factor involved in many inflammation pathways including signaling downstream of TNF [52]. (3) CLI-095 that blocks TLR4 [56], which is a receptor and signaling pathway that is upstream of many pro-inflammatory cytokines/chemokines such as TNF and KC [57]. (4) Glyburide that inhibits NALP3, which is a component of the inflammasome and coded by the NLRP3 gene [58]. (5) Indomethacin that blocks COX1/2, which is a critical pathway leading to prostaglandin production [40]. Parthenolide was the only inhibitor that reversed PAH-induced dysfunction of GJIC in the C10 cells at 24 h, but not 4 h. In E10 cells, the GJIC response with parthenolide was similar to the C10 cells at 24 h, however at 4 h, parthenolide exposure in combination with the PAHs was close but not significantly different than PAH treatment (Figure 3c,d). PAH metabolism could be involved after 8 h, but not prior to 8 h as these LMW PAHs are not metabolized in C10 cells indicating that the cellular events before 8 h are functions of the non-metabolized parent compounds [40]. However, PAH byproducts might be involved at later times in which ~50–60% of the PAHs are metabolized at 24 h [40]. Thus, this suggests that the PAH metabolites or the metabolites in conjunction with the remaining parent compounds are eliciting these effects on GJIC at 24 h, further supported by the parthenolide-induced reversal of PAH-induced CX43 protein repression (Figure 5). Collectively, it appears that a more pan-inflammation inhibition of primary known pathways that lead to pro-inflammatory cytokine and chemokine production are necessary to elicit effects on gap junctions following PAH exposures because none of the specific inhibitors had any effect on gap junctions in C10 or E10 cells (see Figure 11).

To provide evidence that cytokines can act more directly in dysregulating gap junctions in our model, we chose a PAH-induced cytokine, TNF, observed in the epithelial and macrophage cells (Figure 2, Figure 7 and Figure S3). TNF is a pro-inflammatory cytokine with known tumor promoting and tumor repressing properties studied in many types of cancers, including lung, and is involved in both acute and chronic inflammation [51]. This cytokine is downstream of both NFκB and AP-1 transcription factor pathways, and thus is also downstream of TLR4 signaling [51]. Once TNF binds to its receptors (TNFR1 and TNFR2), the NFκB pathway is triggered, which in turn activates the NALP3 inflammasome [59]. Thus, this cytokine is connected to the pathways inhibited via parthenolide in our model system. Additionally, TNF heterozygote (−/+) mice were found to develop less lung tumors than wildtype control mice in urethane-induced lung cancer [60] and TNF is in a critical chromosomal loci for both cancer and many lung inflammatory diseases [25], further supporting a role of TNF in the development of lung disease and cancer. Our novel data using rTNF provides the needed evidence that cytokines can act alone to trigger the dysfunction of GJIC in lung epithelial cells at relevant doses in addition to providing evidence that these PAHs that persist in the cells can act together with cytokines to elicit adverse effects on lung cells (see Figure 9 and Figure 10). Figure 11 depicts the findings from these studies in lung cells.

Other groups have determined the effects of rTNF in liver and lung alveolar type II cell lines [53,61,62]. In WB liver cells, an oval cell or stem-like cell line, rTNF alone elicited dysregulation of GJIC only after 24 h at similar doses to those used in our studies (1–20 ng/mL). These authors did not observe any alterations in GJIC prior to 24 h with rTNF, but only assessed a 1 h time point, whereas we examined a 4 h time point in the C10 cells and did see significant dysregulation (~20%) at all doses tested. LMW PAHs were also individually used (fluoranthene, phenanthrene, and pyrene) in the WB cells, however, these LMW PAHs were not found to further exacerbate the rTNF-induced dysregulation of GJIC at 24, unlike our findings in lung at lower PAH doses [53]. We also used the lowest rTNF dose (1 ng/mL) to represent a more physiological dose in our model and probe for potential interactions with PAH-induced responses on GJIC and Cx43 protein expression, which is also why we used a lower dose (8 μM) of the LMW PAH mixture (Figure 9 and Figure 10). These doses were also lower than the doses used in the liver cell model suggesting the potential that the lung cells are not as metabolically active at 24 h (noted above and in [40]), and that our parent compounds are still influencing the observed responses at 24 h. In addition to affecting gap junctions, rTNF was shown to work alone or in coordination with B[*a*]P (a HMW PAH) to upregulate CYP1B1 in a rat alveolar type II cell line (RLE-6TN), potentially increasing the metabolism of procarcinogens and therefore playing a role in early tumor development [61]. Lastly, rTNF combined with B[*a*]P in these RLE-6TN cells also increased reactivity with DNA as indicated by B[*a*]P-induced DNA adduct formation potentially leading to genotoxic events. Additionally, TNF increased mRNA production of inflammatory mediators, namely *iNos*, *Cox2*, *Il6*, and *Il1β* [62], some of which were cytokines detected in our studies. However, the dose of rTNF used for these RLE-6TN cell line studies was 20 ng/mL [61,62] and therefore the physiological relevance of this dose comes into question.

We previously published data that provided evidence that the LMW PAH mixture elicited a co-carcinogenic effect when combined with B[*a*]P in the C10 cells [35]. In these studies, we observed significant increases in DNA adduct formation resulting from the primary B[*a*]P metabolite (BPDE) combined with the same two LMW PAHs in our mixture used for the present studies. In addition, we observed significant increases in GJIC dysregulation with the B[*a*]P and LMW PAH combination, as well as an increase in COX-2 mRNA expression, all supporting early stage tumor promotion associated phenotypes. However, in these studies we did not directly assess promotion per se because we applied the toxicants at the same time and thus concluded that at the least, these LMW PAHs should be considered co-carcinogens. However, currently, they are considered non-toxic, non-genotoxic, and non-carcinogenic in many cellular models [50,63,64]. DNA adduct formation is a hallmark of cancer described by Hanahan and Weinberg (2011) that contributes to genome instability and mutations [28], and is a key step during the initiation stage of multistage cancer processes. However, other important events that occur during tumor promotion involve both gap junction dysfunction and inflammation.

Gap junction dysregulation and dysfunction is part of the evasion of growth suppression and the current hypothesis is that gap junctions can act as tumor suppressors, at least in early cancer development [29]. Connexins and gap junctions in lung epithelial cells are involved in homeostatic functions such as the regulation of surfactant secretion in type II cells, calcium signaling between type I and II cells, ciliary beating, and other cell signaling events [42]. A disruption in these functions could lead to adverse effects and more injury in these cells, such as alterations in growth and the potential link to acute lung injury or acute respiratory distress syndrome (ARDS) that requires cell-cell communication [42,65]. In addition, idiopathic pulmonary fibrosis patients had decreased CX43 expression [38] and a mouse model with both *Cx40*^−/−^ and *Cx43*^−/−^ (endothelial) KO mice had fibrosis like symptoms 8 wks after birth [66]. However, it is currently unclear how to account for the other research supporting a pro-inflammatory role for gap junctions in the lung [67]. Interestingly, the liver may be a potential clue where in a chronic liver model, increases in CX43 expression are associated with inflammation; when CX43 is blocked in this liver model, the liver damage is far worse suggesting the CX43 expression is involved in an adaptive protective response [68].

Tumor promoting inflammation is also a hallmark of cancer [28] and our laboratory and many others have established a role for inflammatory pathways, such as TLR4, TNF, NFκB, among many others, in lung cancer development [25,60,69,70]. TLR4 is a complicated pathway; in acute lung inflammation/injury models, TLR4 is typically pro-inflammatory [71,72], whereas in lung cancer, appears to be protective [73]. In astrocytes, CX43 was degraded in response to acute LPS treatment [74], while in C10 cells, bronchoalveolar lavage from *Tlr4*^−/−^ mice inhibited gap junctions and CX43 expression compared to wildtype mice [75]. Thus, it depends on acute versus chronic as to the involvement of the TLR4 pathway and the link to gap junctions.

Lastly, there are several future directions for our research. These studies did not investigate other lung expressed connexins, such as CX26 and CX32, the possibility of hemichannel involvement, nor tight junction protein involvement, all of which have potential roles in our LMW-PAH induced lung model [42]. Cross talk between tight junctions and connexins is commonly observed in lung [42], with specific proteins like ZO-1 and CX43 directly interacting. Collectively, these novel studies addressing the association between inflammation and gap junctions in an environmentally relevant model provide additional empirical evidence for future risk assessments on these LMW PAHs to prevent adverse health effects as well as identifying potential new therapeutic targets, such as connexins, for early stage lung cancer.

## 4. Materials and Methods

### 4.1. Materials and Reagents

Parthenolide (pan-inflammation inhibitor: NFκB, AP-1, and NALP3 inflammasome pathway inhibitor) and ACHP (NFκB specific inhibitor) were purchased from TOCRIS (0610, 4547, Bristol, UK). Indomethacin (COX1/2 inhibitor) was purchased from Sigma-Aldrich (I7378, St. Louis, MO, USA). CLI-095 (TLR4 inhibitor) was purchased from InvivoGen (tlrl-cli95, San Diego, CA, USA). Fluoranthene was obtained from AccuStandard (H-118N, purity 97.2%, New Haven, CT, USA) and 1-methylanthracene from Crescent Chemical (DRE-C20834900, purity 99.5%, Islandia, NY, USA). Glyburide (Glybenclamide; NLRP3 inhibitor) was purchased from Novus (NBP2-30141, Centennial, CO, USA). All inhibitors and PAH were dissolved in DMSO (Thermo Fischer Scientific, Waltham, MA, USA), as done previously [36,37,40]. Recombinant mouse TNF-alpha (rTNF) was purchased from R&D systems (410-MT, Minneapolis, MN, USA).

### 4.2. Cell Line Maintenance and Experimental Design

Both the C10 and E10 cell lines, immortalized non-transformed alveolar type II cell lines, were obtained as a kind gift from Dr. Lori Nield (University of Colorado, Denver Anschutz Medical Center, CO, USA). These cell lines were originally derived from BALB mice [76,77] and are ideal for our studies because they are from a progenitor cell type known to initiate non-small cell lung carcinomas [78,79]. Cells were maintained in CMRL 1066 media (Thermo Fisher Scientific) containing 10% fetal bovine serum, 1% L-glutamine, and 1% Penicillin Streptomycin. Both C10 and E10 cells were grown to confluency and serum deprived for 24 h prior to treatment. C10 and E10 cells were pre-treated with inhibitors 1 h before treatment with 40 μM or 15 μM binary PAH mixture for 4 h and 24 h, respectively. Our LMW binary PAH mixture consisted of a 1:1 ratio of two prevalent LMW PAHs found environmentally and in secondhand smoke, 1-methylanthracene and fluoranthene. These doses and ratio for PAH exposure were used previously in the C10 cells in our lab and are the IC_50_ for dysregulation of GJIC at 4 h (40 μM) or 24 h (15 μM) [36,37,40]. For the E10 cells, following cytotoxicity evaluation, we performed dose response studies for gap junction activity to determine doses, as was done previously with the C10 cells and determined similar IC50s to the C10 cells at both times, thus we used the same doses for the E10 cells.

MHS cells are an alveolar macrophage cell line originally derived from BALB/c mice [80]. MHS cells were maintained in RPMI media with 2 mM L-glutamine, 1% Penicillin Streptomycin, 10% FBS, 2.38 g HEPES (10 mM), 10 µL sodium pyruvate (1 mM), 2.25 g glucose, 0.75 g sodium bicarbonate, and 1.75 μL 2-mercaptoethanol. Cells were grown to confluency and serum deprived for 24 h prior to treatment. Cells were treated with ≤20 μM of the binary PAH mixture, a dose determined to be non-cytotoxic.

### 4.3. Cytotoxicity

Cytotoxicity was assessed in all cell lines using the CellTiter 96 Aqueous One Solution Cell Viability assay (MTS assay, Promega, Madison, WI, USA) following manufacturer’s instructions. Cells were grown in 96 well cell culture plates and serum deprived for 24 h prior to treatment with PAH, pharmaceutic inhibitors, rTNF, or their combinations.

### 4.4. Scalpel Loaded/Dye-Transfer Assays to Measure Gap Junctional Intercellular Communication (GJIC)

GJIC activity was determined using the method described by Upham et al. (2011) [81]. C10 cells were grown to confluency and treated as noted above. Immediately following the treatment, cells were washed three times with PBS and in the presence of a Lucifer Yellow dye (1 mg/mL in PBS), three cuts were made with a steel scalpel blade. The dye was allowed to transfer between gap junctions for 3 min. The cells were then washed again with PBS three times and fixed with 4% formalin in PBS. The cut lines were imaged with an Eclipse TI-S microscope at 100× and captured using a DS-QiMc camera (Nikon Instruments, Melville, NY, USA). The area of dye spread, considered GJIC, was quantified using ImageJ software (http://imagej.nih.gov/ij/). Treatment groups were compared to DMSO, the vehicle control, for final fraction of control (FOC) percentages. Three images were taken of each individual line, three cuts were made per dish, and there were three dishes per treatment, for a total of *n* = 9.

### 4.5. KC Measurement via ELISA

KC protein levels were evaluated using the mouse CXCL1/KC DuoSet ELISA (DY453, R&D) following manufacturer’s instructions. Cells were washed with saline before a 1 h treatment with inhibitor and a 4 h PAH treatments. At sample addition, the conditioned media retrieved was diluted 1:1 with reagent diluent (5% BSA in PBS) and incubated overnight in 96 well ELISA plate. The plate was read at 450 nm using the Tecan Infinite M200 Pro plate reader (Morrisville, NC, USA).

### 4.6. CX43 Protein Expression Using Immunoblots

Protein from C10 cells was extracted using 20% SDS containing Halt Protease & Phosphatase Single-Use Inhibitor Cocktail (Pierce, Waltham, MA, USA), similar to Osgood et al. (2013 and 2017, [36,37]). Twenty µg of protein per sample were separated on a 12.5% SDS page gels and transferred to a polyvinylidene fluoride (PVDF) membrane (Millipore, Burlington, MA, USA). Anti-rabbit Cx43 antibody (cat# 3512s, Cell Signaling, Danvers, MA, USA) and anti-rabbit B-actin were diluted 1:1000 in Odyssey Blocking Buffer (cat# 927-50000, in 1× TBS) and incubated overnight at 4 degrees Celsius. Secondary antibodies used were IRDye800 CW goat anti-rabbit (1:10,000) for Cx43 and IRDye680 LT goat anti-mouse (1:20,000) for β-actin. Proteins were visualized using the Odyssey Imaging system (Licor, Lincoln, NE, USA) and quantified by densitometry using the Image Studio software (Licor).

### 4.7. Quantitative Reverse Transcriptase PCR (qRT-PCR)

One microgram of total RNA was reverse transcribed to cDNA [37] and amplified with gene-specific primers labeled with Power SYBR Green master mix (Applied Biosystems, Foster City, CA, USA) using an QuantStudio 3 Real time PCR (Applied Biosystems). Samples were normalized to the expression of 18S rRNA using the comparative CT method [82]. Sequences for the primers (*Il1b, Tlr4, Nalp3)* can be found in Table S1 or in previously published works (*Kc*, *Mcp1*, *Cox2*, *Il6*, *18S*) [36,37,40].

## 5. Conclusions

Our studies herein provide novel evidence that GJIC inhibition can be reversed in response to an anti-inflammatory inhibitor in lung epithelial cells. In addition, we show for the first time that a pro-inflammatory cytokine, such as TNF, can influence or potentially interact with an environmental toxicant, such as the PAHs, in lung epithelial cells to inhibit gap junction function as well as CX protein expression. Since we provide evidence that TNF originates from both epithelial cells or macrophages, this supports that both autocrine and paracrine mechanisms are involved. In the future, we will further investigate the mechanisms driving these observed responses, evaluate additional pathways for identification of biomarkers of exposure, and potential new therapeutic targets.

## Figures and Tables

**Table 1:** MHS cell inflammatory cytokines/chemokine response following 4 h treatment with the binary PAH mixture.

PAH Dose * (μM)	*Cox2*		*Tnf*		*Kc*		*Mcp1*		*Il6*		*Il1β*	
	Ave	SEM	Ave	SEM	Ave	SEM	Ave	SEM	Ave	SEM	Ave	SEM
**0**	1.02	0.13	1.01	0.03	1.01	0.11	1.00	0.06	1.04	0.19	1.00	0.07
**5**	1.70	0.15	1.51	0.11	1.37	0.35	1.22	0.07	1.33	0.38	2.01	0.35
**10**	1.02	0.09	1.41	0.19	1.28	0.20	1.55	0.42	1.72	0.35	2.79	0.30 **
**15**	2.30	0.44 **	2.46	0.30 **	1.81	0.32	1.97	0.16	1.73	0.12	5.42	0.16 **
**20**	2.18	0.11 **	2.78	0.25 **	3.09	1.13 **	2.02	0.31	3.20	0.67 **	6.26	0.16 **

* Binary PAH mixture (1:1 ratio of 1-methylanthracene and fluoranthrene); 0 is DMSO only. ** *p* < 0.05 compared to 0 (control; DMSO). Cox2, cyclooxygenase 2; Tnf, tumor necrosis factor; Kc, Cxcl1, keratinocyte chemoattractant; Mcp1, Ccl1, monocyte chemoattractant protein 1; and IL6, interleukin 6.

## Supplementary Materials

The following are available online at https://www.mdpi.com/2072-6694/11/4/572/s1. Figure S1: Representative images for the other inhibitor combinations with 4 h PAH treatment in C10 and E10 cells, Figure S2: Representative images for the other inhibitor combinations with 24 h PAH treatment in C10 and E10 cells, Figure S3: *Tnf* transcript expression in C10 and E10 cells is inhibited by parthenolide in the presence of PAH mixture treatment, Table S1: Primer sequences for qRTPCR analysis.

## Abbreviations

COX2	cyclooxygenase 2
CX	connexin
GJIC	gap junctional intercellular communication
HMW	high molecular weight
KC	keratinocyte chemoattractant; CXCl1
MCP1	monocyte chemoattractant protein 1
LMW	low molecular weight
PAH	polycyclic aromatic hydrocarbon
TNF	tumor necrosis factor alpha
rTNF	recombinant TNF

## References

[1] Lee H.L., Hsieh D.P., Li L.A. (2010). Polycyclic aromatic hydrocarbons in cigarette sidestream smoke particulates from a Taiwanese brand and their carcinogenic relevance. Chemosphere.

[2] Moir D., Rickert W.S., Levasseur G., Larose Y., Maertens R., White P., Desjardins S. (2008). A comparison of mainstream and sidestream marijuana and tobacco cigarette smoke produced under two machine smoking conditions. Chem. Res. Toxicol..

[3] ATSDR (2005). Toxicology Profile for Polyaromatic Hydrocarbons.

[4] Schick S.F., Farraro K.F., Perrino C., Sleiman M., van de Vossenberg G., Trinh M.P., Hammond S.K., Jenkins B.M., Balmes J. (2013). Thirdhand cigarette smoke in an experimental chamber: Evidence of surface deposition of nicotine, nitrosamines and polycyclic aromatic hydrocarbons and de novo formation of NNK. Tob. Control.

[5] IARC (2016). Outdoor Air Pollution.

[6] World Health Organization (2016). Ambient Air Pollution: A Global Assessment of Exposure and Burden of Disease.

[7] IARC (2012). Personal Habits and Indoor Combustions.

[8] D’Amato G., Baena-Cagnani C.E., Cecchi L., Annesi-Maesano I., Nunes C., Ansotegui I., D’Amato M., Liccardi G., Sofia M., Canonica W.G. (2013). Climate change, air pollution and extreme events leading to increasing prevalence of allergic respiratory diseases. Multidiscip. Respir. Med..

[9] IARC (2010). Some Non-Heterocyclic Polycyclic Aromatic Hydrocarbons and Some Related Exposures.

[10] U.S.E.P.A. (2002). Polycyclic aromatic hydrocarbons, 15 listings. Rep. Carcinog..

[11] Severson R.F., Snook M.E., Higman H.C., Chortyk O.T., Akin F.J., Freudenthal R.I., Jones P.W. (1976). Isolation, identification, and quantification of polynuclear aromatic hydrocarbons in tobacco smoke. Carcinogenesis—A comprehensive Survey. Vol. 1. Polynuclear Aromatic Hydrocarbons: Chemistry, Metabolism, and Carcinogenesis.

[12] Hong W.J., Jia H., Ma W.L., Sinha R.K., Moon H.B., Nakata H., Minh N.H., Chi K.H., Li W.L., Kannan K. (2016). Distribution, Fate, Inhalation Exposure and Lung Cancer Risk of Atmospheric Polycyclic Aromatic Hydrocarbons in Some Asian Countries. Environ. Sci. Technol..

[13] Zhang P., Chen Y. (2017). Polycyclic aromatic hydrocarbons contamination in surface soil of China: A review. Sci. Total Environ..

[14] Marczynski B., Pesch B., Wilhelm M., Rossbach B., Preuss R., Hahn J.U., Rabstein S., Raulf-Heimsoth M., Seidel A., Rihs H.P. (2009). Occupational exposure to polycyclic aromatic hydrocarbons and DNA damage by industry: A nationwide study in Germany. Arch. Toxicol..

[15] Pesch B., Kappler M., Straif K., Marczynski B., Preuss R., Rossbach B., Rihs H.P., Weiss T., Rabstein S., Pierl C. (2007). Dose-response modeling of occupational exposure to polycyclic aromatic hydrocarbons with biomarkers of exposure and effect. Cancer Epidemiol. Biomark. Prev.

[16] Talaska G., Thoroman J., Schuman B., Kafferlein H.U. (2014). Biomarkers of polycyclic aromatic hydrocarbon exposure in European coke oven workers. Toxicol. Lett..

[17] Serdar B., Brindley S., Dooley G., Volckens J., Juarez-Colunga E., Gan R. (2016). Short-term markers of DNA damage among roofers who work with hot asphalt. Environ. Health.

[18] Allan S.E., Smith B.W., Anderson K.A. (2012). Impact of the deepwater horizon oil spill on bioavailable polycyclic aromatic hydrocarbons in Gulf of Mexico coastal waters. Environ. Sci. Technol..

[19] Bojes H.K., Pope P.G. (2007). Characterization of EPA’s 16 priority pollutant polycyclic aromatic hydrocarbons (PAHs) in tank bottom solids and associated contaminated soils at oil exploration and production sites in Texas. Regul. Toxicol. Pharm..

[20] Fustinoni S., Campo L., Cirla P.E., Martinotti I., Buratti M., Longhi O., Foa V., Bertazzi P. (2010). Dermal exposure to polycyclic aromatic hydrocarbons in asphalt workers. Occup. Environ. Med..

[21] Dubey J., Banerjee A., Meena R.K., Kumari K.M., Lakhani A. (2014). Characterization of polycyclic aromatic hydrocarbons in emissions of different mosquito coils. Bull. Environ. Contam. Toxicol..

[22] Lung S.C., Hu S.C. (2003). Generation rates and emission factors of particulate matter and particle-bound polycyclic aromatic hydrocarbons of incense sticks. Chemosphere.

[23] Salvi S.S., Barnes P.J. (2009). Chronic obstructive pulmonary disease in non-smokers. Lancet.

[24] Szewczyńska M., Pośniak M., Dobrzyńska E. (2012). Study on the individual PAHs content in ultrafine particles from solid fractions of diesel and biodiesel exhaust fumes. J. Chem..

[25] Bauer A.K., Malkinson A.M., Kleeberger S.R. (2004). Susceptibility to neoplastic and non-neoplastic pulmonary diseases in mice: Genetic similarities. Am. J. Physiol..

[26] Klaunig J.E., Kamendulis L.M., Xu Y. (2000). Epigenetic mechanisms of chemical carcinogenesis. Hum. Exp. Toxicol..

[27] Malkinson A.M. (1998). Molecular comparison of human and mouse pulmonary adenocarcinomas. Exp. Lung Res..

[28] Hanahan D., Weinberg R.A. (2011). Hallmarks of cancer: The next generation. Cell.

[29] Nahta R., Al-Mulla F., Al-Temaimi R., Amedei A., Andrade-Vieira R., Bay S.N., Brown D.G., Calaf G.M., Castellino R.C., Cohen-Solal K.A. (2015). Mechanisms of environmental chemicals that enable the cancer hallmark of evasion of growth suppression. Carcinogenesis.

[30] Trosko J.E. (2001). Commentary: Is the concept of “tumor promotion” a useful paradigm?. Mol. Carcinog..

[31] Trosko J.E., Chang C.C. (1986). Oncogene and chemical inhibition of gap-junctional intercellular communication: Implications for teratogenesis and carcinogenesis. Prog. Clin. Biol. Res..

[32] Trosko J.E., Chang C.C., Madhukar B.V., Dupont E., Iversen O.H. (1993). Oncogenes, tumor suppressor genes and intercellular communication in the ‘Oncogeny as partially blocked ontogeny’ hypothesis. New Frontiers in Cancer Causation.

[33] Spath C., Schlegel F., Leontyev S., Mohr F.W., Dhein S. (2013). Inverse Relationship between Tumor Proliferation Markers and Connexin Expression in a Malignant Cardiac Tumor Originating from Mesenchymal Stem Cell Engineered Tissue in a Rat in vivo Model. Front. Pharm..

[34] Avanzo J.L., Mesnil M., Hernandez-Blazquez F.J., Mackowiak I.I., Mori C.M., da Silva T.C., Oloris S.C., Garate A.P., Massironi S.M., Yamasaki H. (2004). Increased susceptibility to urethane-induced lung tumors in mice with decreased expression of connexin43. Carcinogenesis.

[35] Bauer A.K., Velmurugan K., Plottner S., Siegrist K.J., Romo D., Welge P., Bruning T., Xiong K.N., Kafferlein H.U. (2017). Environmentally prevalent polycyclic aromatic hydrocarbons can elicit co-carcinogenic properties in an in vitro murine lung epithelial cell model. Arch. Toxicol..

[36] Osgood R.S., Upham B.L., Hill T., Helms K.L., Velmurugan K., Babica P., Bauer A.K. (2013). Polycyclic aromatic hydrocarbon-induced signaling events relevant to inflammation and tumorigenesis in lung cells are dependent on molecular structure. PLoS ONE.

[37] Osgood R.S., Upham B.L., Bushel P.R., Velmurugan K., Xiong K.N., Bauer A.K. (2017). Secondhand Smoke-Prevalent Polycyclic Aromatic Hydrocarbon Binary Mixture-Induced Specific Mitogenic and Pro-inflammatory Cell Signaling Events in Lung Epithelial Cells. Toxicol. Sci..

[38] Trovato-Salinaro A., Trovato-Salinaro E., Failla M., Mastruzzo C., Tomaselli V., Gili E., Crimi N., Condorelli D.F., Vancheri C. (2006). Altered intercellular communication in lung fibroblast cultures from patients with idiopathic pulmonary fibrosis. Respir. Res..

[39] Avanzo J.L., Mesnil M., Hernandez-Blazquez F.J., da Silva T.C., Fukumasu H., Mori C.M., Yamasaki H., Dagli M.L. (2006). Altered expression of connexins in urethane-induced mouse lung adenomas. Life Sci..

[40] Siegrist K.J., Romo D., Upham B.L., Armstrong M., Quinn K., Vanderlinden L., Osgood R.S., Velmurugan K., Elie M., Manke J. (2019). Early Mechanistic Events Induced by Low Molecular Weight Polycyclic Aromatic Hydrocarbons in Mouse Lung Epithelial Cells: A Role for Eicosanoid Signaling. Toxicol. Sci..

[41] Guan X., Hardenbrook J., Fernstrom M.J., Chaudhuri R., Malkinson A.M., Ruch R.J. (1995). Down-regulation by butylated hydroxytoluene of the number and function of gap junctions in epithelial cell lines derived from mouse lung and rat liver. Carcinogenesis.

[42] Johnson L.N., Koval M. (2009). Cross-talk between pulmonary injury, oxidant stress, and gap junctional communication. Antioxid. Redox Signal..

[43] Chanson M., Berclaz P.Y., Scerri I., Dudez T., Wernke-Dollries K., Pizurki L., Pavirani A., Fiedler M.A., Suter S. (2001). Regulation of gap junctional communication by a pro-inflammatory cytokine in cystic fibrosis transmembrane conductance regulator-expressing but not cystic fibrosis airway cells. Am. J. Pathol..

[44] Vikis H.G., Gelman A.E., Franklin A., Stein L., Rymaszewski A., Zhu J., Liu P., Tichelaar J.W., Krupnick A.S., You M. (2012). Neutrophils are required for 3-methylcholanthrene-initiated, butylated hydroxytoluene-promoted lung carcinogenesis. Mol. Carcinog..

[45] Gong L., Cumpian A.M., Caetano M.S., Ochoa C.E., De la Garza M.M., Lapid D.J., Mirabolfathinejad S.G., Dickey B.F., Zhou Q., Moghaddam S.J. (2013). Promoting effect of neutrophils on lung tumorigenesis is mediated by CXCR2 and neutrophil elastase. Mol. Cancer.

[46] Gong L., da Silva Caetano M., Cumpian A.M., Daliri S., Garza Flores A., Chang S.H., Ochoa C.E., Evans C.M., Yu Z., Moghaddam S.J. (2016). Tumor necrosis factor links chronic obstructive pulmonary disease and K-ras mutant lung cancer through induction of an immunosuppressive pro-tumor microenvironment. Oncoimmunology.

[47] Alexander C.M., Xiong K.N., Velmurugan K., Xiong J., Osgood R.S., Bauer A.K. (2016). Differential innate immune cell signatures and effects regulated by toll-like receptor 4 during murine lung tumor promotion. Exp. Lung Res..

[48] Allavena P., Mantovani A. (2012). Immunology in the clinic review series; focus on cancer: Tumour-associated macrophages: Undisputed stars of the inflammatory tumour microenvironment. Clin. Exp. Immunol..

[49] Fritz J.M., Dwyer-Nield L.D., Malkinson A.M. (2011). Stimulation of neoplastic mouse lung cell proliferation by alveolar macrophage-derived, insulin-like growth factor-1 can be blocked by inhibiting MEK and PI3K activation. Mol. Cancer.

[50] Upham B.L., Blaha L., Babica P., Park J.S., Sovadinova I., Pudrith C., Rummel A.M., Weis L.M., Sai K., Tithof P.K. (2008). Tumor promoting properties of a cigarette smoke prevalent polycyclic aromatic hydrocarbon as indicated by the inhibition of gap junctional intercellular communication via phosphatidylcholine-specific phospholipase C. Cancer Sci..

[51] Balkwill F. (2009). Tumour necrosis factor and cancer. Nat. Rev. Cancer.

[52] Cho H.Y., Morgan D.L., Bauer A.K., Kleeberger S.R. (2007). Signal transduction pathways of tumor necrosis factor--mediated lung injury induced by ozone in mice. Am. J. Respir. Crit. Care Med..

[53] Kabatkova M., Svobodova J., Pencikova K., Mohatad D.S., Smerdova L., Kozubik A., Machala M., Vondracek J. (2015). Interactive effects of inflammatory cytokine and abundant low-molecular-weight PAHs on inhibition of gap junctional intercellular communication, disruption of cell proliferation control, and the AhR-dependent transcription. Toxicol. Lett..

[54] Juliana C., Fernandes-Alnemri T., Wu J., Datta P., Solorzano L., Yu J.W., Meng R., Quong A.A., Latz E., Scott C.P. (2010). Anti-inflammatory compounds parthenolide and Bay 11-7082 are direct inhibitors of the inflammasome. J. Biol. Chem..

[55] Saadane A., Eastman J., Berger M., Bonfield T.L. (2011). Parthenolide inhibits ERK and AP-1 which are dysregulated and contribute to excessive IL-8 expression and secretion in cystic fibrosis cells. J. Inflamm. (Lond.).

[56] Ii M., Matsunaga N., Hazeki K., Nakamura K., Takashima K., Seya T., Hazeki O., Kitazaki T., Iizawa Y. (2006). A novel cyclohexene derivative, ethyl (6R)-6-[N-(2-Chloro-4-fluorophenyl)sulfamoyl]cyclohex-1-ene-1-carboxylate (TAK-242), selectively inhibits toll-like receptor 4-mediated cytokine production through suppression of intracellular signaling. Mol. Pharm..

[57] Kawai T., Akira S. (2011). Toll-like receptors and their crosstalk with other innate receptors in infection and immunity. Immunity.

[58] Lamkanfi M., Mueller J.L., Vitari A.C., Misaghi S., Fedorova A., Deshayes K., Lee W.P., Hoffman H.M., Dixit V.M. (2009). Glyburide inhibits the Cryopyrin/Nalp3 inflammasome. J. Cell Biol..

[59] He Y., Hara H., Nunez G. (2016). Mechanism and Regulation of NLRP3 Inflammasome Activation. Trends Biochem. Sci..

[60] Bernert H., Sekikawa K., Radcliffe R.A., Iraqi F., You M., Malkinson A.M. (2003). Tnfa and Il-10 deficiencies have contrasting effects on lung tumor susceptibility: Gender-dependent modulation of IL-10 haploinsufficiency. Mol. Carcinog..

[61] Smerdova L., Smerdova J., Kabatkova M., Kohoutek J., Blazek D., Machala M., Vondracek J. (2014). Upregulation of CYP1B1 expression by inflammatory cytokines is mediated by the p38 MAP kinase signal transduction pathway. Carcinogenesis.

[62] Umannova L., Machala M., Topinka J., Schmuczerova J., Krcmar P., Neca J., Sujanova K., Kozubik A., Vondracek J. (2011). Benzo[a]pyrene and tumor necrosis factor-alpha coordinately increase genotoxic damage and the production of proinflammatory mediators in alveolar epithelial type II cells. Toxicol. Lett..

[63] Ghoshal B., Weber W.J., Rummel A.M., Trosko J.E., Upham B.L. (1999). Epigenetic Toxicity of a Mixture of Polycyclic Aromatic Hydrocarbonson Gap Junctional Intercellular Communication Before and After Biodegradation. Environ. Sci. Technol..

[64] Tai M.H., Upham B.L., Olson L.K., Tsao M.S., Reed D.N., Trosko J.E. (2007). Cigarette smoke components inhibited intercellular communication and differentiation in human pancreatic ductal epithelial cells. Int. J. Cancer.

[65] Koffler L., Roshong S., Kyu Park I., Cesen-Cummings K., Thompson D.C., Dwyer-Nield L.D., Rice P., Mamay C., Malkinson A.M., Ruch R.J. (2000). Growth inhibition in G(1) and altered expression of cyclin D1 and p27(kip-1)after forced connexin expression in lung and liver carcinoma cells. J. Cell. Biochem..

[66] Chatterjee S., Baeter S., Bhattacharya J. (2007). Endothelial and epithelial signaling in the lung. Am. J. Physiol..

[67] Parthasarathi K., Ichimura H., Monma E., Lindert J., Quadri S., Issekutz A., Bhattacharya J. (2006). Connexin 43 mediates spread of Ca2+-dependent proinflammatory responses in lung capillaries. J. Clin. Investig..

[68] Balasubramaniyan V., Dhar D.K., Warner A.E., Vivien Li W.Y., Amiri A.F., Bright B., Mookerjee R.P., Davies N.A., Becker D.L., Jalan R. (2013). Importance of Connexin-43 based gap junction in cirrhosis and acute-on-chronic liver failure. J. Hepatol..

[69] Dwyer-Nield L.D., Srebernak M.C., Barrett B.S., Ahn J., Cosper P., Meyer A.M., Kisley L.R., Bauer A.K., Thompson D.C., Malkinson A.M. (2005). Cytokines differentially regulate the synthesis of prostanoid and nitric oxide mediators in tumorigenic versus non-tumorigenic mouse lung epithelial cell lines. Carcinogenesis.

[70] Xiao Z., Jiang Q., Willette-Brown J., Xi S., Zhu F., Burkett S., Back T., Song N.Y., Datla M., Sun Z. (2013). The pivotal role of IKKalpha in the development of spontaneous lung squamous cell carcinomas. Cancer Cell.

[71] Bauer A.K., Rondini E.A., Hummel K.A., Degraff L.M., Walker C., Jedlicka A.E., Kleeberger S.R. (2011). Identification of candidate genes downstream of TLR4 signaling after ozone exposure in mice: A role for heat-shock protein 70. Environ. Health Perspect..

[72] Zhang X., Shan P., Qureshi S., Homer R., Medzhitov R., Noble P.W., Lee P.J. (2005). Cutting edge: TLR4 deficiency confers susceptibility to lethal oxidant lung injury. J. Immunol..

[73] Bauer A.K., Upham B.L., Rondini E.A., Tennis M.A., Velmuragan K., Wiese D. (2017). Toll-like receptor expression in human non-small cell lung carcinoma: Potential prognostic indicators of disease. Oncotarget.

[74] Liao C.K., Jeng C.J., Wang H.S., Wang S.H., Wu J.C. (2013). Lipopolysaccharide induces degradation of connexin43 in rat astrocytes via the ubiquitin-proteasome proteolytic pathway. PLoS ONE.

[75] Hill T., Osgood R.S., Velmurugan K., Alexander C.M., Upham B.L., Bauer A.K. (2013). Bronchoalveolar Lavage Fluid Utilized Ex Vivo to Validate In Vivo Findings: Inhibition of Gap Junction Activity in Lung Tumor Promotion is Toll-Like Receptor 4-Dependent. J. Mol. Biomark. Diagn..

[76] Malkinson A.M., Dwyer-Nield L.D., Rice P.L., Dinsdale D. (1997). Mouse lung epithelial cell lines—Tools for the study of differentiation and the neoplastic phenotype. Toxicology.

[77] Bentel J.M., Lykke A.W., Smith G.J. (1989). Cloned murine non-malignant, spontaneously transformed and chemical tumour-derived cell lines related to the type 2 pneumocyte. Cell Biol. Int. Rep..

[78] Desai T.J., Brownfield D.G., Krasnow M.A. (2014). Alveolar progenitor and stem cells in lung development, renewal and cancer. Nature.

[79] Lin C., Song H., Huang C., Yao E., Gacayan R., Xu S.M., Chuang P.T. (2012). Alveolar type II cells possess the capability of initiating lung tumor development. PLoS ONE.

[80] Mbawuike I.N., Herscowitz H.B. (1989). MH-S, a murine alveolar macrophage cell line: Morphological, cytochemical, and functional characteristics. J. Leukoc. Biol..

[81] Upham B.L. (2011). Role of integrative signaling through gap junctions in toxicology. Curr. Protoc. Toxicol..

[82] Bauer A.K., Velmurugan K., Xiong K.N., Alexander C.M., Xiong J., Brooks R. (2017). Epiregulin is required for lung tumor promotion in a murine two-stage carcinogenesis model. Mol. Carcinog..

