# Supplementary material for: Dysregulation of Gap Junction Function and Cytokine Production in Response to Non-Genotoxic Polycyclic Aromatic Hydrocarbons in an In Vitro Lung Cell Model
Source: Cancers (Basel). 2019 Apr 23;11(4):572. doi: 10.3390/cancers11040572 (PMC6521202; doi:10.3390/cancers11040572)
Supplement: Supplementary file 1 [file cancers-11-00572-s001.pdf]

Supplementary Materials

Dysregulation of Gap Junction Function and Cytokine Production in Response to Non-Genotoxic Polycyclic Aromatic Hydrocarbons in an In Vitro Lung Cell Model

Deedee Romo, Kalpana Velmurugan, Brad L. Upham, Lori D. Dwyer-Nield and Alison K. Bauer

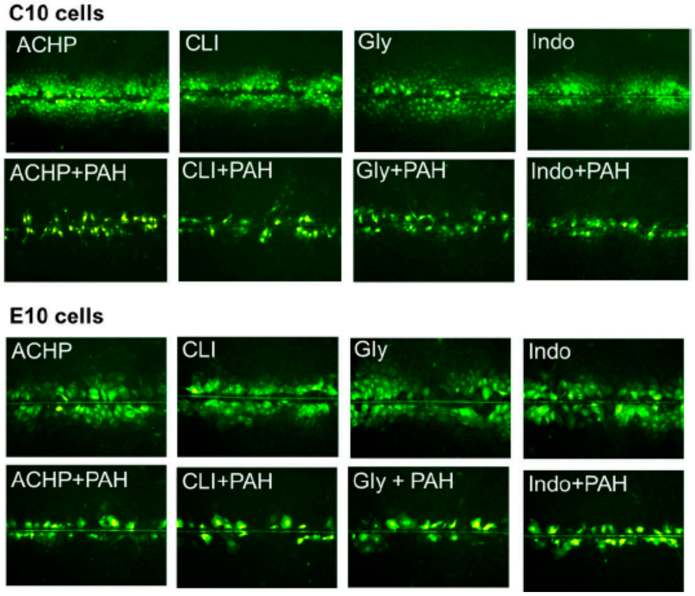

Figure S1. Representative images for the other inhibitor combinations with 4 h PAH treatment in C10 and E10 cells. C10 and E10 cells were treated with the binary LMW PAH mixture (40 mM at 4 h; 1:1 ratio of 1-methylanthracene and fluoranthene) for 4 h following 1 h pre-incubation with these inhibitors (ACHP, 1 mM; CLI-095, 3 mM; glyburide, 50 mM; indomethacin, 1 mM). Representative images of C10 and E10 cells following the SL/DT assay used to quantify the gap junction activity in these cells in response to the LMW PAHs and ACHP, CLI-095, or indomethacin combinations. Experiments were repeated 3 times; *n* = 3 per treatment per experiment. Gly, glyburide; Indo, indomethacin. The control and PAH alone treatments are found in Figure 3.

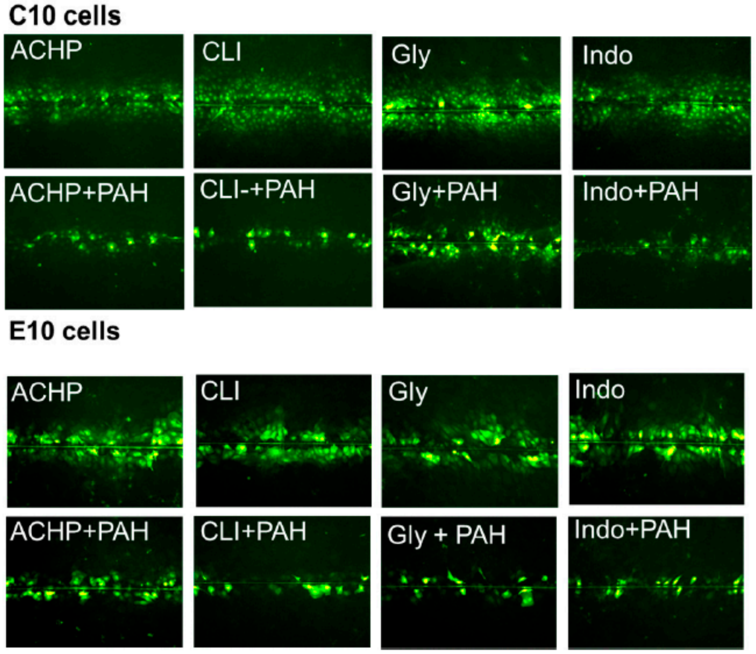

Figure S2. Representative images for the other inhibitor combinations with 24 h PAH treatment in C10 and E10 cells. C10 and E10 cells were treated with the binary LMW PAH mixture (15 mM at 24 h; 1:1 ratio of 1-methylanthraceneand fluoranthene) following a 1 h pre-incubation with these inhibitors (ACHP, 1 mM; CLI-095, 3 mM; glyburide, 50 mM; indomethacin, 1 mM). Representative images of C10 and E10 cells following the SL/DT assay used to quantify the gap junction activity in these cells in response to the LMW PAHs and all inhibitor combinations, except parthenolide. Experiments were repeated 3 times; n = 3 per treatment per experiment. Gly, glyburide; Indo, indomethacin. The control and PAH alone treatments are found in Figure 4.

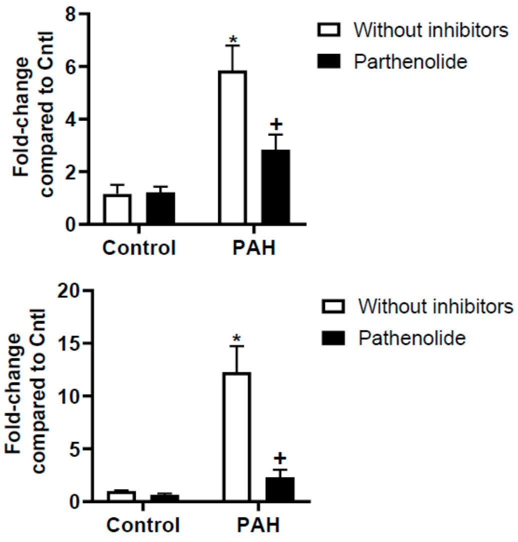

Figure S3. *Tnf* transcript expressionin C10 and E10 cells is inhibited by parthenolide in the presence of PAH mixture treatment. C10 and E10 cells were treated for 4 h with binary LMW PAH mixture (40 mM; 1:1 ratio of 1-methylanthracene and fluoranthene) following a 1 h pre-incubation with parthenolide (10 mM). qRTPCR was then performed for TNF, normalized to 18S followed by comparison to control treated cells (DMSO). Experiments were repeated twice with n = 3 per experiment. * $p < 0.05$ for treatment compared to control; $^{+}$ $p < 0.05$ for PTL + PAH compared to PAH treatment.

Table S1. Primer sequences for qRTPCR analysis *.

Name		Primer Sequence	Genbank Accession/NCBI
Il1β	Forward (5′→3′)	CAA CCA ACA AGT GAT ATT CTC CAT G	M15131.1
	Reverse (5′→3′)	GAT CCA CAC TCT CCA GCT GCA	
NALP3	Forward (5′→3′)	CTC TAT CAA GGA CAG GAA CG	Nm_145827.3
	Reverse (5′→3′)	TAG GAT GGT TTT CCC GAT GC	
TLR4	Forward (5′→3′)	ATT GAA GAC AAG GCA TGG CAT	AF095353
	Reverse (5′→3′)	GTG AGC CAC ATT GAG TTT CTT	

* Kc, Cox-2, Il6, Mcp-1, 18S primers are published in Osgood et al., 2017 [37]; Tnf primers are published in Osgood et al, 2013 [36].

© 2019 by the authors. Licensee MDPI, Basel, Switzerland. This article is an open access article distributed under the terms and conditions of the Creative Commons Attribution (CC BY) license (http://creativecommons.org/licenses/by/4.0/).
